# Supplementary material for: Are carbon emissions trading and green financial instruments synergistic? -Comprehensive quantitative research based on content analysis
Source: PLoS One. 2024 Mar 7;19(3):e0298601. doi: 10.1371/journal.pone.0298601 (PMC10919601; doi:10.1371/journal.pone.0298601)
Supplement: S1 Data — (ZIP) [file pone.0298601.s001.zip › Data/地级市Z_F工作报告.zip!/╣π╢1⁄2/╓╨╔╜╩╨/└┤╫╘╣1⁄2╓┌║┼í╛┬φ┐╦╩2╛▌═°í┐_2003.docx]

各位代表：

我代表市人民政府向大会作政府工作报告，请予审议，并请政协各位委员和其他列席人员提出意见。

2002年工作回顾

2002年，是我市经济社会各项事业持续快速健康发展的一年。一年来，在省委、省政府和中山市委的领导下，在市人民代表大会及其常务委员会和市政协的监督支持下，市政府坚持以邓小平理论和“三个代表”重要思想为指导，根据中央“稳定、安全、灵活、多元”的经济工作方针，按照“三个进一步提高、两个增强”的工作方略，动员全市人民以率先基本实现社会主义现代化为总目标、总任务，紧紧把握加入世贸的机遇，大力推进“工业强市”、“科教兴市”、“依法治市”和“可持续发展”战略的实施，全面实现了市十一届人大四次会议确定的国民经济和社会发展预期目标。

综合经济实力进一步增强。实现国内生产总值415.5亿元，比上年增长（下称增长）16.5%，增速居全省第2位。投资、出口、消费三大需求活跃，全年固定资产投资211.7亿元，增长19%。财税增长，累计入库税收77.5亿元，增长23.9%。地方财政一般预算收入31.2亿元，一般预算支出34.9亿元（可比口径），分别增长34.5%和30.6%。金融形势平稳，中小金融机构再贷款的偿还和企业集资款兑付按计划进行，年末各项存款余额和贷款余额分别比年初增长19.3%和19%。

工业强市取得新成效。全市实现工业总产值1344亿元，增长28.4%；工业增加值224.7亿元，增长21.7%；新办工业企业1200多家。继续推进工业集约发展，加快工业园区建设步伐，积极扶持区域特色经济，调整优化产业结构，抓好名牌战略和技术创新，全年技改投入19.7亿元，完成技改项目105个，有18个工业产品获得国家、省的免检或名牌产品称号，9个产品获得省著名商标称号。工业生产保持快速增长的势头。成功举办了3.28招商经贸洽谈会、武汉（中山）博览会、（沙溪）国际休闲服装节、（古镇）国际灯饰博览会，（中山）国际电子信息技术与产品博览会等一系列会展活动，签定贸易合同210亿元。我市获得了国家有关部门授予的“全国纺织工业基地市”称号，小榄、古镇、沙溪分别获得“中国五金制品产业基地”、“中国灯饰之都”、“中国休闲服装名镇”称号，进一步改善了我市的产业环境，提高了工业产品的市场竞争力。企业经营的宏观环境和微观环境同步改善，景气度继续回升，工业产销衔接良好，产品销售率达97.5%，综合效益指数提高了9.5个百分点。支柱产业的龙头作用更加突出，五大支柱产业产值增长30.3%，高于工业平均增长水平1.9个百分点。

农业和农村工作取得新进展。高度重视农业的基础地位，坚持“多予、少取、放活”，以增加农民收入为中心，提高农业整体素质为重点，农产品种养、加工和流通并举，以农业产业化促进了农业经济效益的稳步提高。全年农业总产值32.34亿元，增长7.8%。进一步推进农村“五项工程”，加强水利设施和农业现代化示范区的建设，“五项工程”总投入2.54亿元，建成农路392.7公里，农桥211座，整治内河涌土方321.48万立方米，整治低产鱼塘3500多公顷，乡村“四旁”绿化种植457公里，山地种果230多公顷。高度重视农村稳定工作，切实加强农村财务管理，全面铺开农村税费改革、农村股份合作制改革。行政村调整工作取得阶段性成效。

第三产业稳步增长。出台了《进一步加快第三产业发展的若干意见》，第三产业准入门槛逐步降低，连锁超市、物流配送等现代流通业态迅速发展，一批知名的物流企业先后落户我市；市民消费层次显著提高，住房、汽车、教育、文化、体育、信息等消费活跃。通过三次调减电价，实现了城乡同网同价；实行农村税费改革以及教育收费“一费制”等措施，直接减轻企业及群众负担10多亿元，有效地拉动了消费需求。全年社会消费品零售总额134.3亿元，增长12.4%；旅游业持续快速发展，全年总收入55.38亿元，增长22.3%。房地产业稳步发展。

对外经贸取得新的突破。加快建立以世贸规则相适应的政策体系，废止了100多个与世贸规则不相适应的政策性文件，大力营造亲商、安商、富商氛围。积极实施科技兴贸和市场多元化战略，市政府通过加大实施出口退税账户托管、财政贴息等措施，支持外贸企业扩大出口。全年实现外贸出口57.25亿美元，增长31.3%。利用外资稳步增长，新批外商投资企业386宗，合同利用外资12.54亿美元，实际利用外商直接投资7.68亿美元，分别增长35.4%、26.4%和19.5%。

城市集聚和承载功能进一步增强。进一步加大了城市基础设施的投入，共安排8亿多元，启动了人民桥重建、翠山路等建设工程，建成了城南路、长江北路、东明北路、景观路等一批城市基础设施；加大力度实行城市管理重心下移，城市管理得到进一步加强；共有10个镇被命名为省级“卫生镇”；继续实施“小城镇大战略”，城市化水平稳步提高，镇村建设取得了新的成绩。

“科教兴市”迈出新步伐。新建工程技术研究开发中心和企业技术中心35个，初步形成了多层次的产学研结合的科技创新体系。在各个专业镇组建了工程技术研究开发中心，6个镇区进入省科技创新专业试点镇的行列。高新技术产业不断发展，全年高新技术产品产值占工业总产值的17.5%。在全省年度科技创新能力评估中，我市位居第5。进一步整合教育资源，调整学校布局，改造了一批薄弱学校；教育教学质量进一步提高；电子科技大学中山学院正式开办，广东药学院中山校区筹办工作顺利进行。

各项改革不断深化。加大审批制度改革力度，再次减少审批事项362项。公有资产管理、运营体系不断完善，受托运营的考核类公有企业利润增长12.62%。事业单位机构改革取得阶段性成果，机构和编制分别减少了12.65%和20.55%，全面实行了公开招考录用公务员制度，疏通人才准入渠道，通过引进、培养，各级各类人才总量达到19万人。

人民生活水平不断提高。城镇居民人均可支配收入14208元，增长11%；农民人均年纯收入5872元，增长11.2%。社保体系进一步完善，全年各类险种参保人数达220多万人次，社会保障覆盖面进一步扩大；各级财政增加了资金投入，提高了低保对象的保障标准，基本实现了“应保尽保”。高度重视城乡就业问题，继续完善扶持性政策，城镇登记失业率控制在2.5%以下；解决“双困户”住房共1.89万平方米；旧城改造总面积达9万多平方米；城市环境综合定量考核继续居于全省前列，市民的生活质量进一步改善。

依法行政水平有新的提高。坚持重大事项向人大常委会报告制度，市政府领导与人大常委会领导、政协委员座谈会制度；一批关系国计民生的重大事项按法定程序报请市人大常委会审定；政府决策的民主化和科学化水平有所提高。教育收费“一费制”等与市民关系密切的公共事务，在决策前实行了“听证制度”。进一步转变政府职能，按照“小政府、大社会”的管理模式，进行“简政放权”，全面推行行政执法责任制和过错追究制度，大力促进政府效率“提速”，努力塑造廉洁勤政高效务实的政府形象。大兴调查研究之风，机关作风和公务员队伍建设得到加强。全市党政机关信息统一平台全面建成开通，政务信息化取得新进展。深入开展“严打”斗争和各项专项整治，各类重大案件得到有效遏制。积极预防并妥善处理群体性事件，社会大局稳定。“两项整治”成效显著。统一部署和开展了联合打假，整顿文化市场、建筑市场，强化税收征管，开展对集贸市场、加油站以及旅游市场打假等专项行动，查办案件共4千多宗，市场经济秩序进一步向好。安全生产形势稳定。

各项社会事业有新的发展。深入开展了学习“三个代表”重要思想和“十六大报告”活动，为经济社会的健康发展提供了强大的精神动力。大力开展精神文明创建活动，进一步提高了广大市民的思想道德文化水平和民主法治观念。社会科学和文化艺术领域涌现出一批高质量的精神产品，获得一批国家级和省级奖项，提高了城市的文化品位。新闻、出版事业健康发展。全民健身活动蓬勃开展，在省第十一届运动会上我市取得总分和金牌总数第三名的好成绩。医疗服务质量不断提高，社区卫生服务人口覆盖率达72.4%；城乡卫生面貌不断改善。计划生育形势保持平稳，全年常住人口出生率为9.51‰，计划生育率为92%，完成了省下达的人口控制指标。

监察、审计、统计、老干、侨务、外事、信访、档案、地方志、妇女儿童、民族宗教、台湾事务、国防建设、老区建设等工作也取得了新成绩。

各位代表，2002年我市经济和社会发展取得的成就，是中共广东省委、省政府和市委正确领导，全市人民共同努力的结果。在此，我代表市人民政府向全市工人、农民、知识分子、干部、驻我市人民解放军指战员、武警部队官兵和公安干警，表示崇高的敬意！向全市人民，各民主党派，各人民团体和其他各界人士对政府的信任和支持，表示衷心的感谢！向港澳台同胞、海外侨胞和所有关心支持参与中山建设的国内外友人表示诚挚的谢意！

回顾一年的工作，我们也清醒地看到经济社会发展中还存在不少困难和问题。主要是经济增长的内在动力不足，投资推动型的增长模式仍占主导地位；结构调整未能适应经济快速发展的要求，镇区之间发展不够平衡；城市功能型、枢纽型的基础设施建设与高速增长的经济和社会发展需求不相适应；转变政府职能，改进机关作风，提高服务水平和效率等仍需作出更大的努力。这些问题需要我们在今后的工作中努力加以解决。

2003年政府工作的总体安排

2003年是贯彻党的十六大精神，全面建设小康社会的第一年，做好今年的各项工作意义十分重大。从国际看，当前国际形势处于复杂深刻的变动之中，世界多极化和经济全球化趋势在曲折中发展，国际经济虽有复苏迹象，但不确定因素仍很多。与此同时，我们还要看到世界性产业结构调整和国际资本流动也为我们提供了有利的机遇。从国内看，党的十六大对全面建设小康社会作出了总体部署，明确了社会经济发展的目标和任务。我国经济将承接前几年发展的良好态势，进入一个新的发展阶段。同时，加入世贸的正面效应逐步显现，积极财政政策和扩大内需的拉动效应进一步加强。从全省看，省九次党代会提出建设经济强省，加快率先基本实现社会主义现代化的战略目标，为今后的发展指明了方向。面对新形势，我们必须保持清醒的头脑，进一步增强机遇意识、忧患意识和责任意识，与时俱进，开拓创新，充分调动全市各方面力量，全力以赴做好各项工作。

2003年政府工作的总体要求是：认真贯彻落实党的十六大、省九次党代会和市委十届六次全会精神，以全面建设小康社会，率先基本实现社会主义现代化为总目标、总任务统揽全局，按照“三个进一步提高、两个增强”的工作方略，继续抢抓机遇，以提高城市综合竞争力为核心，积极参与区域分工；加快经济结构调整，坚持走新型工业化道路，促进三大产业协调发展；继续深化改革，增创开放、产业、科技、体制、环境五大优势，进一步增强发展活力，促进经济和社会各项事业的健康发展；切实加强民主法制建设，促进社会主义物质文明、政治文明和精神文明协调发展。

2003年我市经济社会发展的主要预期目标是，国内生产总值增长13%；工业产值增长18%，工业增加值增长16%；全社会固定资产投资增长13%，社会消费品零售总额增长12%；外贸出口总值增长10%，实际利用外资增长8%；城镇居民人均可支配收入增长7%；农民人均年纯收入增长5%；城镇登记失业率控制在3.5%以下；人口自然增长率控制在5.4‰以内。

为实现上述预期目标，必须切实做好以下几项工作：

一、走新型工业化道路，全面增强制造业竞争力

大力推进经济结构调整，推动产业升级。继续坚定不移推进“工业强市”战略，全面提升工业发展的水平。加快与国际产业结构大调整，国际资本大流动的对接。逐步建立以高新技术产业开发区和健康医药产业基地为龙头，支柱产业为骨干，以专业基地、区域经济为特色，以工业园区为依托的制造业体系，努力实现从一般加工基地向高级加工基地以及服务基地的转变。主动接受周边城市优势产业的辐射。以发展电子信息、生物医药、新能源、新材料、光机电一体化等新兴产业带动全市工业体系实现质的飞跃。着力营造产业氛围，延伸产业链条，进一步凸现支柱产业的优势。集中力量建设好国家级健康医药科技产业基地。完善产业政策，加快产业集聚，积极吸引国际国内医药企业到基地落户，加快基地的产业化、国际化步伐。

以信息化带动工业化。加快信息技术在传统产业、区域特色产业中的应用，积极利用先进实用技术改造提升传统产业，进一步加快有自主知识产权和核心技术的信息产品的研制和开发，认真落实国家、省和我市鼓励软件、集成电路产业发展的政策，全面提升信息制造业的竞争力。依托大型企业，积极发展电子商务。千方百计增加工业产品的科技含量和附加值。

开展工业示范园区建设，实现园区错位发展。加快对现有园区在资源、项目上的整合，全面提高园区建设标准，镇区要集中资金，加快已立项园区各项基础设施的建设；全面开展工业示范园区建设，使园区形成更强的承载国际产业资本、促进产业集聚能力。市有关部门要加强宏观管理，为园区发展提供规划导向、建设指南和行政服务，促进园区的有序竞争。按照产业分工的特点，促进园区错位发展，形成产业梯度分布。搞好“银园”合作。引入外资、民资，调整土地开发、经营思路，将园区基础设施作为产业来发展。

培育核心竞争力，提高企业经济效益。鼓励企业做大做强，培育企业适应国际市场竞争的规模经营能力，技术创新和管理创新能力；注重人力资源开发，提高资本经营能力和企业科学管理水平，实现管理创新。要把增强企业的品牌效应作为培育企业核心竞争力的重要内容，提高企业自主知识产权开发能力，形成自己的核心技术，培育名牌产品和著名商标。

加快区域特色经济发展。充分发挥区域特色优势和族群产业优势，制定长远规划，完善产业导向，加大政策扶持，办好各类专业会展，进一步凸显产业特色。建立健全科技创新体系，通过政府扶持和引导，积极探索特色镇技术创新服务中心的经营运作机制。支持一批对特色经济发展有重要影响的关键技术和共性技术的引进和开发，不断提升产品质量、技术含量和效益水平。积极探索专业镇发展新模式，着力经营好国家级的中国电子（中山）基地、纺织工业基地、五金制品产业基地、灯饰之都、健康产业基地、包装印刷基地、精细化工基地、中国休闲服装名镇；争取申报亚洲包装中心、红木家具制造基地等一批国家级、世界级的制造业基地；鼓励镇区依托市场，提高产业的聚合和辐射能力，形成新的区域品牌。

二、以农民增收为目标，加快发展农业和农村经济

加大农业结构调整力度。大力发展农业优势产品，培育主导产业和拳头产品，力争在引进、推广名、优、新、特、稀品种上有新的突破。完善农业区域经济布局。按标准化生产的要求，积极开展农业标准的制定，抓好优质农产品认证和标识工作。大力发展无公害产品和“绿色食品”，全面提高农产品质量安全水平。建立市、镇及农产品市场三级农产品质量安全检验检测体系，确保食品安全。要加快农业产业化步伐。推动农业向生产、加工、销售一体化经营转变，提高农业的整体素质和效益。加大搞活农产品流通加工的力度，建立健全农产品的流通队伍和市场体系，抓好国内外市场的开发，引进农产品精、深加工项目，进一步把农产品流通加工推向一个新水平。要加大力度扶持农业龙头企业的发展，探索龙头企业上规模、上效益的新路子。扶持农业各种专业协会或行业组织的发展，提高农业的组织化程度。大力发展农业信息服务、农业科技咨询与指导、生资供应、机耕作业服务等配套服务，逐步建立农业的产前、产中、产后系列化服务体系。全面提高农业机械化生产水平，抓好农业现代化示范区建设，加快农业现代化进程。

加强农业和农村基础设施建设。全面推进农村“五项工程”，实行市、镇、村三级联动，多渠道筹措资金，加快农桥、农路建设，内河整治，低产鱼塘整治，绿化和“一区三线”森林改造工程，改善农业生产条件。要抓好整体规划，实行综合治理，把“五项工程”的建设与生产布局调整、村镇建设、环境治理等结合起来，充分发挥综合效益。加强水利基础设施建设，做好险堤、险段整治及中小型水库的除险加固，提高防灾、抗灾能力。

大力发展农村经济，努力增加农民收入。确立以城镇建设促经济集聚，促社会进步的观念，围绕产业发展和镇区经济发展特点，处理好建设、环保、资源的关系，建设一批基础性、功能性工程，促进全市城镇建设整体水平有明显提高。进一步发挥中心镇、中心村的辐射和带动作用，加快城镇化步伐。通过调整农村产业结构、加快农村劳动力向二、三产业转移。全面实行涉农价格公示和教育收费“一费制”，确保农民增收。发展壮大镇村集体经济，高度重视和解决好区域经济发展不平衡问题。加大财政转移支付的力度，设立专项贷款贴息资金，扶持经济欠发达镇区的发展；切实做好镇村增资减债工作。

深化农村改革，确保农村大局稳定。巩固和发展行政村调整成果，切实理顺行政村调整后的各种关系。积极稳妥推进农村股份合作制改革。进一步理顺农村集体经济的产权关系、利益分配关系，推动土地、劳动力等生产要素的合理配置和加快流转。要高度重视解决农民转移就业、基层干部作风、经济和财务管理、征地款分配等“热点”问题，各级领导要深入到农民群众当中，倾听他们的意见、呼声和要求，把各种潜在矛盾和问题解决在基层和萌芽状态。

三、加快城市建设步伐，完善城市功能

加快东部沿海开发建设和四大组团发展战略研究。按照互补、错位、共享的原则，积极参与珠三角、粤港澳城际分工和经济合作，明确城市区域分工和产业定位，主动承接城市圈的部分城市功能和产业转移，当好配角，加快与大珠三角经济带接轨，从提升城市综合竞争力的战略需要出发，抓紧制定和修编总体发展战略、概念规划和开发建设的阶段目标，加快区域性中心城市的建设步伐。抓紧与国道、省道、轻轨等建设规划的衔接，从功能设置、产业布局、基础设施等方面整合资源，优化配置，全面加快基础设施、配套设施、功能性设施的建设。进一步拓展城市发展空间，加快中部、西北部、东部沿海、南部“四大组团”的规划和建设，完善以中心城区为第三产业密集区，“一区一园三基地”为高新技术密集区，南部为出口加工区和特色工业镇、各类工业园区为依托的比较合理的产业空间布局，加快形成中心城区到东部沿海的经济带。

完善市域交通网络，营造“半小时生活圈”。强化城市承载功能和集聚能力，进一步拉开城市框架。全面启动“五纵五横”交通主干线的规划和建设。加快城桂路二期、翠山公路建设；建设古神公路；加快新岐江公路、东环路、南沙公路等工程立项论证工作。加强镇际公路规划建设，形成贯通全市，畅顺快捷的市域交通网络。市财政安排1000万元，全面启动危桥（涵）改造工程。加大旧城区改造和城市主干道出入口整治的力度，多角度、全方位美化、绿化城市，凸现城市特色。启动市文化艺术中心、会展中心、紫马岭南片综合小区、长堤商业区、岐江广场等项目建设，加快能源、通讯等基础设施建设。建设逸仙、古镇同益、环城旗乐等220千伏变电站。通过城市基础设施的高强度投入，不断提高城市品位和文化内涵，打造“精品”城市。

加大力度经营城市，拓宽城市建设的投入渠道。以市场为主、政府为辅，多渠道筹集资金，加大城市建设投入力度。采取招标、租赁、抵押等方式，出让部分市政公用设施经营权；放宽准入限制，引入市场机制，鼓励外资、民资投入基础设施建设；支持国家、省驻市机构投资建设。组建城乡建设投融资公司和土地储备中心，建立土地储备制度，整理回收闲置土地，管好用好非农用地指标，通过市场化运作凸现城市土地、环境的潜在价值。加快“城中村”改造。继续推动城市管理重心下移，通过政策的、市场的推动，进一步提高城市公共产品的水平，使城市建设更加充满活力。

四、扩大有效需求，全面提升第三产业发展水平

大力发展现代服务业，提高第三产业对经济增长的贡献率。依托区位优势和特色经济优势，加快制定物流业发展规划，引导物流业有序发展。加快形成社会化、专业化现代物流体系，推动传统物流的现代化，努力把我市建设成为区域性物流基地。以中山港为依托，发展港口货物运输相关行业的国际物流园区；以区域特色产业为依托，建设区域工业运输、仓储相关行业的产业配送型物流园区；以商品配送为导向，以满足我市和周边城市生产和生活需要为目标，发展以配送服务为主体的城市配送型物流园区。加快各类专业市场建设。重点建设东升花木市场、民众沙仔香蕉批发市场及小榄五金制品、沙溪纺织服装、古镇灯饰、大涌家具、东风小家电、黄圃食品等特色产品批发市场。加强物流园区与专业批发市场的衔接，促进各类特色专业批发市场向专业物流中心转变。采取政府推动、第三方实施、市场化运作的模式，加快建立覆盖政府职能部门和商业、贸易、金融、制造等领域，以产品生产、运输、储存、配送等环节的信息流为基础、具有增值服务功能的物流信息平台。要大力发展现代服务业，提高服务业的技术含量和对经济增长的贡献。加快旅游业发展，提升“国家优秀旅游城市”形象。积极探索休闲度假旅游、会展旅游、都市旅游、特色产业旅游等新型旅游方式。鼓励和扶持大型旅游景点和设施的建设。加快培育一批主业突出，经营规模大，具有区域性竞争力的大型连锁企业，不断推动连锁经营向电脑软件、汽车配件、房地产以及家政等新兴行业发展。加快金融、证券、保险业的发展；发展技术、信息、咨询、法律、会计等中介服务业以及社区服务业。在规范管理的基础上，对一些专营性、垄断性、资源性项目的经营，要积极引入市场机制。要进一步释放民间资本、外来资本对第三产业的投资需求。

千方百计扩大消费需求。把扩大内需同经济结构的战略性调整、深化经济体制改革、增加就业、改善人民生活和可持续发展结合起来。进一步培育和扩大内需，努力增加城乡居民的收入。引导和扩大文化消费、教育消费、体育消费、旅游消费、高科技消费等各种新的消费方式，拓宽信贷领域，鼓励市民充分享受现代文明成果。金融等部门要大力推动信用体系的建设，为群众旅游、购车、购房等大宗消费提供多层次、多样化的消费信贷服务，提升市民的消费预期。加大力度对国内市场进行深度开拓。有条件的镇区，要积极推动建立以区域特色经济为依托的全国销售和服务网络，发挥区域特色经济在拓展国内市场空间方面的整体优势。发挥会展对经济发展的拉动作用，打造会展业的名牌，扩大会展的影响力和竞争力。

五、积极参与国际竞争，全面提高对外开放水平

优化外贸出口结构，大力培育与国际标准接轨的出口工业体系。要按照世贸的规则，促进出口企业采用国际标准，加大出口产品生产环节技术改造的投入，提升传统大宗出口产品的质量、档次、科技含量和附加值；鼓励企业打造更多有自主知识产权的出口品牌，培植一批能牵动行业发展的拳头出口产品。不断完善一般贸易出口促进体系，加大对企业进行境外参展、质量认证、倾销应诉、业务培训等的扶持力度；在确保对欧盟、美加、日本、东南亚等重点市场出口的持续稳定增长的同时，积极拓展东欧、中东、拉丁美洲等市场，力争在开拓直接远洋贸易、探索多元化贸易方式、发展一般贸易出口等方面实现新的突破。加大对各类出口企业的扶持力度，大力开展出口退税帐户托管贷款业务，年出口超100万美元的各类出口企业，其出口贷款贴息在享受省的政策的基础上市财政继续给予贴息支持。

提高利用外资的质量。将利用外资与经济结构调整紧密结合起来，鼓励有利于我市重点发展的项目和区域特色经济的项目优先进入，重点加强与世界500强企业的联系和合作。发挥我市工业园区招商引资的载体作用以及产业、行业的聚集效应，吸引更多不同类型的企业特别是具有牵动效应的关联项目进园进区投资设厂，进一步扩大和优化我市的外资存量。积极推动招商引资向社会化、市场化、信息化的运作模式发展，逐步推行代理招商、网上招商、中介招商、文化招商等现代招商方式。探索建立全市联动、协调运作的招商工作机制，把招商引资的重点从单纯吸引资金为主转移到以引进资金和引进先进技术、引进现代化管理、引进专门人才相结合上来。加大高新技术产业、支柱产业、新兴产业和第三产业的引资力度。要充分发挥外事、侨务、台湾事务部门在引资、引智，开展对外经济、文化交流方面的积极作用，进一步扩大开放的领域。

继续改善投资环境。进一步提高“大通关”水平，推动口岸管理工作走向规范化、科学化。建立有效的口岸工作联络协调机制，加强关检、关港、关贸、关企等各方面的合作，推进通关业务改革，通过电子化、网络化、模式化运作，缩短通关时间，降低通关成本，建立通关绿色通道，进一步推动口岸整体效率的提高。

六、全面释放民间资本投资需求，大力发展民营经济

大力营造有利于民营经济发展的社会环境，按照“政治平等、政策公平、法律保障、放手发展”的方针，继续优化政策、政务、法制、市场、舆论环境，进一步放宽民营经济进入领域，不限发展比例，不限发展速度，不限经营方式，不限经营规模，切实降低市场准入门槛。凡是国家没有明令禁止的行业和部门，对外商开放的投资领域，实行特殊优惠政策的领域都适用于民间资本进入。全面简化对民间投资性项目的审批手续。拓宽民营企业融资渠道，研究和解决民营企业“贷款难”问题。以体制、科技、管理创新为核心，进一步提高民营经济的整体素质。鼓励和引导民营企业在发展中加快从分散经营向集约化经营转变，从劳动密集型向劳动密集与高科技相结合型转变。大力培植民营骨干企业，引导有实力的企业以资本扩张和业务合作为手段，通过联合、兼并、收购、合资、合作等方式，实施低成本扩张，尽快形成规模效应。积极扶持中小企业的发展，以政府推动，市场运作的模式，构建中小企业的创业发展平台。支持各类行业协会、商社等各种组织的发展，发挥其在政府与企业之间的中介以及促进行业有序竞争和规范经营行为等方面的积极作用，促进民营企业做大做强。

七、深化各项改革，进一步增强体制活力

继续深化公有企业改革，完善公有资产的管理体系。切实履行公有资产出资人职责，实现公有资产保值增值。继续推进产权制度改革，探索建立和完善权利、义务和责任相统一，管资产和管人、管事相结合的公有资产管理体制。加大力度实施营运机构的实体化运作，进一步加快市属全资、控股企业的改革，加快债务重组以及资产的流动和变现，确保公有资产安全，提高资产运营效率。逐步理顺公用事业管理体制。增强上市公司融资、收益能力，提高对全市经济的拉动作用。通过产权变现、债务重组等方式，减轻债务，盘活沉淀资产，实现净资产收益水平的进一步提高。

加快建立公共财政框架，提高财政资金的使用效益。采取积极措施，确保市与镇区分税分成财政体制改革的顺利实施。全面建立镇区一级预算制度。要深化部门预算改革。进一步完善政府采购管理体制、运行机制和监督体系，扩大政府采购规模和范围，进一步规范政府采购行为。加快推进国库集中支付制度改革，严格收支两条线管理。优化财政支出结构，优化资源配置，提高非经营性资产的使用效率。建立公共财政支出体系，确保财政资金集中用于公共产品的生产。

加快社会信用体系建设，优化市场经济秩序。重点抓好民用爆破器材、废旧物品收购和旧货市场、制售假冒伪劣产品、高压走廊违章建筑等重点整治以及国家和省统一部署的集贸市场、加油站、旅游市场、金融和信用、地方保护以及行业垄断、中介机构、安全生产等专项整治，全面规范特殊行业的管理，确保“两项整治”目标如期实现；大力推进《质量振兴纲要》的实施，全面提高产品标准覆盖率；开展知识产权保护工作，做好商标专利工作，为企业经营营造良好的外部环境。强化税收征管；防范和化解金融风险；全面开展社会“诚信工程”建设，优化营商环境。

八、推进科技、教育创新，增强经济发展后劲

坚持科技工作面向经济建设主战场，提升科技竞争力。积极营造有利于科技项目充分发挥经济和社会效益的运行环境，加强创新体系的建设。优化科技创新的主体和平台，更好地为企业科技研发服务。重点抓好100项技术创新和改造工程，以科技水平的提高来推动产业层次和竞争能力的提升。全面加快“一区一园三基地”的建设，提高产业化水平；鼓励有条件的企业积极参与国家、省的重大科技计划和项目开发；配合以电子信息、健康医药等为龙头的高新技术产业的发展，逐步建立完整、系统的科技培训基地、人才培养基地、科技成果孵化基地和工程研究中心。年内高新技术产品产值占全市工业总产值、高新技术企业总量较上年要有明显提高。积极利用高新技术改造传统产业。以六个省级专业镇为重点，实施全市镇区科技示范工程，扶持镇区进行产业分工、创新平台建设，大力推广电子计算机辅助设计、制造等实用技术；加快以广东（中山）农业科技园区为重点的现代农业科技园区的建设。

继续加大科技投入。建立和完善以政府投入为引导、企业投入为主体、金融机构贷款为支撑、其他投入为补充的多渠道、多形式的全社会科技经费投入体系。市政府计划年内投入科技三项经费2000万元，技改贴息3000万元，支持企业进行科技创新和技术改造。鼓励各类科技风险投资公司前来投资，支持各种对高新技术项目进行技术参股、期权分配；继续完善科技中介服务体系，加快建设技术产权交易所等科技中介机构，推进科技成果的市场化步伐。

推进国民经济和社会信息化。实施智能交通示范工程、金盾工程以及中小学信息教育“校校通”等信息化重点工程。按照“统筹规划、统一标准、互联互通、资源共享”的原则，推进政务信息化建设。全面加快“网上审批”系统建设，实现面向社会的行政审批、管理和服务业务网上运行，年内争取实现一半以上政府备案、核准性事项的“网上申报”和“网上审批”。在完善党政机关信息网络统一平台的基础上，加大资源整合的力度，提高行政效率和服务水平。

把发展教育放在更加突出的位置，进一步提高教育整体水平。积极推进创建“教育强市”和“教育强镇”工作，完善分级办学、分级负责的管理体制，形成市政府统筹规划、宏观调控，镇区政府主动承担、分工落实，村民自治组织积极参与，社会各方大力支持的基础教育办学新格局。增加教育投入，确保税费改革后教育投入高于改革前的水平。进一步整合优化教育资源，调整中小学校布局，扩大学校规模，提高办学质量。不断提高适龄人口高等教育在学率、在校学生计算机的生机比。全面深化教育改革。按照社会和经济发展的需求，调整好普通高中与职业高中的比例，大力推进职业教育。全面推行教育人事制度改革，努力建设一支充满活力的教师队伍。积极发展“民办教育”、职业教育和继续教育，建设学习型社会，逐步建立终身教育体系。

营造优秀人才脱颖而出的良好环境。进一步完善人才引进政策，大力推进“人才市场”的市场化运作，鼓励人才“柔性流动”，最大限度地开发人力资源，为高层次人才进入我市提供各种便利条件。要结合产业结构调整和企业创新的实际，明确人才培育、引进的层次和方向，加大各类人才特别是高层次、复合型、实用型以及科技中介服务型人才引进力度，不断提高人才储备的总量。继续加强企事业单位博士后工作站的建设。做好为非公有制经济发展人才支持的服务。积极配合中国电子科技大学中山学院、广东药学院中山校区的建设。

九、改善民生，建设适宜居住和创业发展的大环境

把满足群众多样化要求，不断提高人民生活水平，促进人的全面发展，作为政府的一项根本任务。把扩大就业作为社会经济发展的重要目标，多渠道扩大就业，确保就业形势的稳定。要进一步引导广大农民转变就业观念，加强就业培训，各级政府要设立农民再就业的指导和中介服务机构，进一步完善和落实有关优惠政策，市级财政安排1600多万元，专项用于就业的培训补贴，引导和促进农村富余劳动力向第二、三产业转移。鼓励企业优先录用本市劳动力，千方百计拓宽农村富余劳动力的就业渠道。继续稳步推进社保的扩面工作，加大社会保险政策法规的宣传力度，增强企业和劳动者的参保意识。强化社保基金的征缴管理和劳动保障监督检查，进一步完善医保各项配套措施的落实和监督管理。积极探索建立农村社会保障体系。规范最低生活保障制度，切实解决困难群众“看病难”、子女“读书难”等问题，确保低保对象享受低保的覆盖面达到100%。强化人民调解、法律服务和法律援助工作。全面启动新一轮住房解困工程。

大力推进城市生态示范区建设，全面提高环境生态水平。以珠江综合整治为契机，全面启动我市水环境综合整治十年规划及水资源综合规划，加快镇区污水处理厂的规划和建设，把我市的水环境保护和水污染治理提高到一个新的水平。积极推进清洁生产，确保6月底前全市工业污染源排放达到国家标准，全面达标率达到80%。加快中心组团以及其它组团的垃圾处理基地建设，推广垃圾分类处理。坚持防治污染与生态建设并重。设立长江库区水源林市级自然生态保护区，积极探索分质供水。积极推进创建国家级“城市生态示范区”的建设；进一步加大环保执法的力度，全面提高全社会的可持续发展意识。

切实加强人口、计划生育、国土资源管理和社会治安综合治理。全面贯彻落实《人口与计划生育法》和广东省《人口与计划生育条例》，加大实施计划生育工作管理“重心下移”，确保实现年度人口与计划生育管理目标。加强国土资源管理，建立土地储备制度，强化滩涂围垦管理，提高土地综合利用效益。高度重视社会治安工作。抓好科技强警以及社会治安综合治理群防群治网络；坚决打击各种危害社会治安的黑恶势力和黄、赌、毒活动，切实加强对出租屋、娱乐场所的管理，努力建设安居乐业的城市环境。安全生产工作要警钟长鸣，常抓不懈，落实各级安全责任制，遏制重大事故的发生。

十、促进各项社会事业全面发展，推进社会主义民主法制和精神文明建设

切实加强思想道德建设。大力推进精神文明建设，努力满足全体市民不断增长的文化需求，为我市的改革开放和现代化建设提供思想保证和精神动力。全面贯彻实施《公民道德实施纲要》，以建设诚信社会为重点，加强社会公德，职业道德和家庭美德教育，广泛深入开展群众性精神文明创建活动以及文明社区建设。

加强社会主义民主法制建设，推进依法治市。高度重视和加强社会主义政治文明建设。实行政府重大决策听证制度，扩大决策听证范围，提高政府决策的民主化和科学化水平。进一步发挥民主党派、工商联、无党派人士参政议政的作用和人民团体的桥梁纽带作用。不断拓宽人民群众参与社会事务管理，监督政府工作的渠道和层面，要尊重人民代表的民主权利，为人民代表履行监督政府工作的职责提供条件和方便。坚持重大事项向人大及其常委会报告制度，加强与人民政协的联系，全面开通人大代表建议办理在线系统，认真办理各项议案、建议和提案。进一步加大政府督办工作力度，确保政令畅通。完善基层信访网络，把“关口前移”落到实处，努力化解各种社会矛盾，进一步密切政群关系。大力开展“四五”普法，稳步推进城市管理综合执法工作，努力营造文明法治、和谐稳定的社会环境。坚持依法行政、从严治政。坚持不懈开展反腐败斗争和廉政建设，提高公务员队伍的整体素质。加强政风建设、廉政建设、职业道德建设，努力建设一支“深怀爱民之心，恪守为民之责，善谋富民之策，多办利民之事”的公务员队伍。要把提高行政效率，降低行政成本，建设“服务型”政府作为优化投资发展环境，密切政群关系的重要内容。进一步规范政府的监督机制、绩效评估机制。积极引导各方面的力量参与社会管理，协调政府各部门发挥对社会管理的宏观调控作用。大力推进政务公开、机关作风评议等行之有效的工作制度。

促进各项社会事业的协调发展和全面进步。大力发展文化产业，进一步繁荣新闻、出版、图书、影视等各类文化市场，活跃群众文化，深入挖掘和开发利用中山历史文化资源，增强城市文化的竞争力。健全多层次的医疗服务体系，规范农村医疗服务行为；健全社区卫生服务网络，提高社区卫生服务的水平；继续搞好药品集中公开招标采购工作，切实降低群众的医药负担。大力发展体育产业，进一步提高全民健身运动和体育竞技水平。落实好各项优抚政策，积极开展创建全国双拥模范城活动。进一步加强国防教育，抓好民兵预备役工作。加快社会福利事业社会化进程，大力发展妇女、儿童、老龄人、残疾人等各项社会事业。

各位代表！党的十六大和省九次党代会为我市经济社会各项事业的进一步发展指明了方向，着眼当前，机遇催人奋进；展望未来，倍感任重道远。我们一定要在省委、省政府和市委的领导下，高举邓小平理论伟大旗帜，全面贯彻落实“三个代表”重要思想，全面落实市委十届六次全会精神，进一步解放思想，与时俱进，开拓创新，为全面建设小康社会和率先基本实现社会主义现代化而努力奋斗！
